# Molecular Detection and Genotyping of *Enterocytozoon bieneusi* in Racehorses in China

**DOI:** 10.3389/fmicb.2019.01920

**Published:** 2019-08-16

**Authors:** Aiyun Zhao, Dongfang Li, Zilin Wei, Ying Zhang, Yushi Peng, Yixuan Zhu, Meng Qi, Longxian Zhang

**Affiliations:** ^1^College of Animal Science, Tarim University, Alar, China; ^2^College of Animal Science and Veterinary Medicine, Henan Agricultural University, Zhengzhou, China; ^3^Equivets, Beijing, China

**Keywords:** *Enterocytozoon bieneusi*, racehorse, prevalence, genotype, zoonotic

## Abstract

*Enterocytozoon bieneusi* is a widely distributed human and animal pathogen. However, few data are available on the distribution of *E. bieneusi* genotypes in racehorses. In this study, 621 fecal specimens were collected from racehorses at 17 equestrian clubs in 15 Chinese cities. *E. bieneusi* was detected via nested polymerase chain reaction (PCR) amplification of the internal transcribed spacer (ITS) gene. The overall infection rate of *E. bieneusi* was 4.8% (30/621). Statistically significant differences were found in the prevalence of this parasite among the equestrian clubs (χ^2^ = 78.464, *df* = 16, *p* < 0.01) and age groups (χ^2^ = 23.686, *df* = 1, *p* < 0.01), but no sex bias was found among the racehorses for the *E. bieneusi* infections (χ^2^ = 1.407, *df* = 2, *p* > 0.05). Ten *E. bieneusi* genotypes were identified, including seven known genotypes (EbpC, EbpA, Peru6, horse1, horse2, CAF1, and TypeIV) and three novel genotypes (HBH-1, SXH-1, and BJH-1). Phylogenetic analysis showed that EbpC, EbpA, Peru6, horse2, CAF1, TypeIV, BJH-1, and SXH-1 belonged to Group 1 of *E. bieneusi*, HBH-1 belonged to Group 2, and horse2 belonged to Group 6. Our findings advance the current knowledge of *E. bieneusi* prevalence and genotypes in racehorses in China.

## Introduction

*Enterocytozoon bieneusi*, an obligate intracellular eukaryotic pathogen, infects a wide variety of vertebrates and invertebrates, including humans (Santín and Fayer, 2011). Clinical symptoms caused by *E. bieneusi* vary depending on the health status of the infected hosts. Asymptomatic infections or self-limiting diarrhea often occur in immunocompetent or healthy individuals, while chronic or life-threatening diarrhea occur in immunocompromised individuals (Didier and Weiss, 2006; Maikai et al., 2012). Most *E. bieneusi* infections in humans result from fecal-oral transmission of spores from infected hosts through contaminated food or water. A foodborne outbreak was reported in Sweden in 2009 (Decraene et al., 2012). Environmentally resistant, infective spores have been detected in various water bodies, including irrigation water, a drinking-source watershed, recreational water, and wastewater from treatment plants, suggesting the possibility of waterborne transmission (Ben et al., 2012; Li et al., 2012; Galván et al., 2013). Because of the clinical and public health importance of *E. bieneusi*, the National Institutes of Health has ranked it on the category B list^1^, and the Environmental Protection Agency has placed it on the microbial contaminant candidate list of concern for waterborne transmission (Didier et al., 2009). To date, based on sequence analysis of the ribosomal internal transcribed spacer (ITS) gene, 474 *E. bieneusi* ITS genotypes have been identified, and 11 phylogenetic groups have been recognized (Li et al., 2019). Group 2 does not include ruminant-specific genotypes, nor does it specify the zoonotic potential of some genotypes (notably BEB4, BEB6, I, and J); thus, Group 2 genotypes are not host-specific (Wang et al., 2018; Li and Xiao, 2019).

Most reports on *E. bieneusi* infections in animals involve cattle, sheep and other livestock, pets, non-human primates and wildlife; however, few reports are available on horses (Thellier and Breton, 2008; Santín et al., 2018; Zhang et al., 2018). In 2010, Santín et al. first reported on *E. bieneusi* infections in horses in Colombia, after which, *E. bieneusi* infections in horses were reported in Algeria, the Czechia, the United States and China (Table 1). To date, 37 *E. bieneusi* genotypes have been identified in horses. To our knowledge, the following eight genotypes have been reported as having infected humans: BEB6, CZ3, CS-4, D, EbpA, EbpC, O, and Peru8 (Leelayoova et al., 2006; Thellier and Breton, 2008; Sak et al., 2011; Wang et al., 2013, 2018; Liu et al., 2017; Table 1).

**TABLE 1:** *Enterocytozoon bieneusi* occurrence and genotype distribution among horses worldwide.

**Country (region)**	**No. Positive/No. examined (%)**	**Genotype (n)**	**References**
Algeria	15/219 (6.8)^a^	**CZ3** (2), **D** (1), horse1 (6), horse2 (1)	Laatamna et al., 2015
China (Xinjiang)	81/262 (30.9)	**BEB6** (9), CS-4 (5), CM7 (2), CM8 (1), CS-1 (1), **D** (1), **EbpA** (20), **EbpC** (21), G (3), horse1 (4), horse2 (2), **O** (4), **Peru8** (1), PigEBITS4 (2), XJH1 (2), ESH-01 (1), XJH3 (1), XJH4 (1)	Qi et al., 2016
China (Sichuan and Yunnan)	75/333 (22.5)	**D** (1), horse1 (13), horse2 (39), SC02 (16), SCH1 (1), SCH2 (1), SCH3 (1), SCH4 (1), YNH1 (1), YNH2 (1)	Deng et al., 2016b
Colombia	21/195 (10.8)	**D** (4), horse1 (13), horse2 (4)	Santín et al., 2010
Czechia	66/377 (17.5)	**D** (34), **EbpA** (2), G (3), horse1 (7), horse2 (8), horse3 (2), horse4 (1), horse5 (1), horse6 (1), horse7 (1), horse8 (1), horse9 (1), horse10 (1), horse11 (2), **WL15**(1)	Wagnerová et al., 2012
Switzerland	0/24 (0)		Breitenmoser et al., 1999
Spain	0/10 (0)		Lores et al., 2002
United States	7/84 (8.3)	horse1 (7)	Wagnerová et al., 2016
Total	265/1504 (17.6)	**BEB6** (9), CS-4 (5), CM7 (2), CM8 (1), CS-1 (1), **CZ3** (2), **D** (41), **EbpA** (22), **EbpC** (21), ESH-01 (1), G (6), horse1 (50), horse2 (54), horse3 (2), horse4 (1), horse5 (1), horse6 (1), horse7 (1), horse8 (1), horse9 (1), horse10 (1), horse11 (2), **O** (4), **Peru8** (1), PigEBITS4 (2), SC02 (16), SCH1 (1), SCH2 (1), SCH3 (1), SCH4 (1), **WL15** (1), XJH1 (2), XJH3 (1), XJH4 (1), YNH1 (1), YNH2 (1)	

*^*a*^Unsuccessful resequencing of five positive specimens. Genotypes detected in humans are shown in bold.*

Horses are common animals worldwide and have been used for leisure activities, sports, and working purposes, including agricultural production, transportation, and military combat. In recent years, horse racing, as one of the oldest known sports, has become more popular in China, and the value of racehorses has increased. However, only one study has been published on *E. bieneusi* infections in racehorses in China, reporting the prevalence of this pathogen in two equestrian clubs in Sichuan at 10.4% (5/48) and 9.6% (5/52; Deng et al., 2016b). To better understand *E. bieneusi* transmission in racehorses in China, we investigated the occurrence of *E. bieneusi* genotypes in racehorses from 17 equestrian clubs.

## Materials and Methods

### Fecal Specimen Collection and DNA Extraction

**FIGURE 1:**
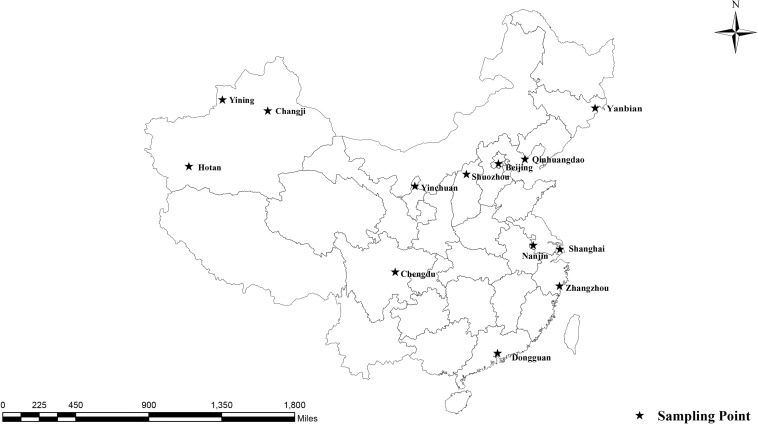
Locations where specimens were collected in this study.

From December 2016 to May 2018, 621 fresh fecal specimens were collected from 30 to 50% of the racehorses at 17 equestrian clubs in 15 Chinese cities (Figure 1 and Table 2). Among the 621 specimens, 74 were from young horses, 547 were from adult horses, 186 were from stallions, 350 were from mares, and 85 were from castrated horses. All horses appeared healthy. Each specimen (30–50 g) was collected directly from the rectum or from the ground immediately after defecation using a sterile disposable latex glove and placed into an individual plastic zip-lock bag. Each individual was identified by the name or number provided by the veterinarians at the equestrian clubs. Specimens were stored in a cooler with ice packs and immediately transferred to the laboratory for testing. The specimens were stored at 4°C, and DNA was extracted within 1 week after collection.

**TABLE 2:** *Enterocytozoon bieneusi* occurrence and genotype profiles in racehorses in China.

**Cities**	**No. Positive/No. samples (%)**	**95% CI**	**χ^2^**	***P-*value**	***E. bieneusi* genotype (n)**
Beijing1	2/32 (6.3)	0–15.1			**Peru6** (2)
Beijing2	0/39				
Beijing3	3/45 (6.7)	0–14.2			**EbpC** (2), BJH-1 (1)
Baicheng	0/29				
Changji	9/34 (26.5)	10.8–42.1			horse1 (9)
Chengdu	1/34 (2.9)	0–8.9			horse1 (1)
Dongguan	1/19 (5.3)	0–16.3			**EbpA** (1)
Hotan	0/21				
Nanjing	0/9	0			
Qinhuangdao	1/136 (0.7)	0–2.2			HBH-1 (1)
Shanghai	2/84 (2.4)	0–5.7			horse1 (2)
Shuozhou	4/15 (26.7)	1.3–52.0			horse1 (3), SXH-1 (1)
Wenzhou	0/23				
Wuhan	3/20 (15.0)	0–32.1			**Peru6** (2), horse2 (1)
Yanbian	0/14				
Yinchuan	0/44				
Zhaosu	4/23 (17.4)	0.6–43.2			horse1 (1), horse2 (1), **TypeIV** (1), **CAF1** (1)
Total	30/621 (4.8)	3.1–6.5	78.464	<0.01	horse1 (16), **Peru6** (4), **EbpC** (2), horse2 (2), **EbpA** (1), **CAF1** (1), **TypeIV** (1), BJH-1 (1), HBH-1 (1), SXH-1 (1)

*Genotypes detected in humans are shown in bold.*

Ten grams of each fecal specimen was thoroughly mixed with 30 mL of distilled water. The suspension was passed through a 250-μm pore wire mesh sieve and centrifuged at 3000 × g for 5 min. The precipitates were used for DNA extraction. Genomic DNA was directly extracted from each (200 mg) precipitate using the E.Z.N.A.^®^ Stool DNA Kit (D4015-02, Omega Bio-tek, Inc., Norcross, GA, United States) per the manufacturer’s instructions with minor modifications. The extracted DNA specimens were stored at −20°C prior to polymerase chain reaction (PCR) analysis.

### PCR Amplification and Sequence Analysis

*Enterocytozoon bieneusi* was detected via nested PCR amplification of the ITS region of the rRNA gene, using the primers and PCR conditions described previously by Buckholt et al. (2002). First, 12.5 μL 2 × EasyTaq PCR SuperMix (TransGene Biotech Co., Beijing, China) was used to amplify each specimen in a 25-μL reaction volume containing 10.9 μL deionized water, 0.3 μM of each primer, 1 μL genomic DNA for the primary PCR, and 1 μL primary amplification product for the secondary PCR. A positive control (DNA from dairy cattle-derived genotype I) and a negative control (distilled water) were used in each PCR run. PCR amplification was repeated twice on each specimen.

All positive secondary PCR amplicons (∼390 bp each) were sent for bidirectional sequencing at GENEWIZ (Suzhou, China). The resultant sequences were assembled using Chromas Pro, version 2.18^2^ and compared with the reference sequences in the National Center for Biotechnology Information^3^ database using ClustalX, version 2.1^4^ to determine the *E. bieneusi* genotypes. The nucleotide sequences obtained in this study were submitted to GenBank^5^ under accession numbers MK789437–MK789446.

### Phylogenetic and Statistical Analyses

The sequences of the ITS regions of the *E. bieneusi* genotypes obtained in this study were compared with those previously identified in humans, other animals and the environment. Bayesian inference (BI) and Monte Carlo Markov chain methods were used to construct phylogenetic trees in MrBayes, version 3.2.6^6^. The general time reversible model (GTR + G) was the best-fit nucleotide substitution model determined by ModelTest, version 3.7^7^. The number of substitutions was set at six, with a proportion of invariable sites. Posterior probability values were calculated by running 1,000,000 generations with four simultaneous tree-building chains. Trees were saved every 1000th generation. At the end of each run, the standard deviation of the split frequencies was <0.01, and the potential scale reduction factor approached one. A 50% majority rule consensus tree was constructed for each analysis using the final 75% of the trees generated via BI. Analyses were run three times to ensure convergence and insensitivity to prior runs. The maximum clade credibility tree generated by these analyses was viewed and edited using FigTree version 1.3.1 software^8^.

The Statistical Package for the Social Sciences (SPSS, version 22.0, available at https://www.ibm.com) was used for the statistical analyses, including Fisher’s exact test and 95% confidence intervals. All results were considered statistically significant at *p* < 0.05.

## Results and Discussion

Among the 621 racehorse fecal specimens analyzed, 30 (4.8%) were positive for *E. bieneusi*, with *E. bieneusi* detected in 10 equestrian clubs (58.8%) from nine cities (60.0%). *E. bieneusi* infection rates were related to the collection sites (χ^2^ = 78.464, *df* = 16, *p* < 0.01; Table 2); the highest infection rates occurred in equestrian clubs in Shuozhou (26.7%, 4/15), Changji (26.5%, 9/34), Zhaosu (17.4%, 4/23) and Wuhan (15.0%, 3/20), with the other sampled equestrian clubs having lower infection rates. Globally, the *E. bieneusi* infection rates in horses range from 0–30.9%. Currently, two studies have reported *E. bieneusi* infections in horses in China: 30.9% (81/262) in Xinjiang (Qi et al., 2016) and 22.5% (75/333) in Sichuan and Yunnan (Deng et al., 2016b), which were higher than those in the racehorses in the present study. This discrepancy may be related to the different management systems used for the horses. Wagnerová et al. (2012) reported that the *E. bieneusi* infection rate in stabled horses (26.6%, 25/94) was higher than that in pastured horses (18.9%, 23/122) and paddocked horses (11.2%, 18/161) in the Czechia. Deng et al. (2016a) reported *E. bieneusi* infection rates in China of 29.1% (48/165) in pastured horses, 26.8% (15/56) in agricultural horses, 10.4% (5/48) in racehorses, and 9.6% (5/52) in equestrian clubs. Racehorses are selected according to their health, are well cared for, and live in good conditions, which may explain their lower *E. bieneusi* infection rates compared with those of other horses.

In the present study, the prevalence rate significantly differed by age group: 16.2% (12/74) in young racehorses and 3.3% (18/547) in adults (χ^2^ = 23.686, *df* = 1, *p* < 0.01; Table 3). Similar findings were reported in a Colombian study, with infection rates of 23.7% (18/76) in young horses and 2.5% (3/119) in adult horses (Santín et al., 2010). However, previous reports from the Czechia and Algeria and from Xinjiang, Sichuan, and Yunnan in China, showed no statistical differences among the age groups of the horses (Wagnerová et al., 2012; Laatamna et al., 2015; Deng et al., 2016b; Qi et al., 2016). Furthermore, no significant differences were found in the prevalence rates among stallions (4.8%, 9/186), mares (5.4%, 19/350) and castrated horses (2.4%, 2/85) (χ^2^ = 1.407, *df* = 2, *p* > 0.05; Table 3). This result is similar to those of previous reports conducted in China, Colombia, Algeria and the Czechia (Santín et al., 2010; Wagnerová et al., 2012; Laatamna et al., 2015; Deng et al., 2016b; Qi et al., 2016). Limited data on this topic suggest that more studies are needed to determine the relationship between age, sex and *E. bieneusi* infections in horses.

**TABLE 3:** *Enterocytozoon bieneusi* occurrence and genotypes among racehorses of different ages and sex.

**Ages and sex**	**No. Positive/No. specimens (%)**	**95% CI**	**χ^2^**	***P-*value**	***E. bieneusi* genotype (n)**
Youths	12/74 (16.2)	7.6–24.8	23.686	<0.01	horse1 (9), horse2 (1), TypeIV (1), CAF1 (1)
Adults	18/547 (3.3)	1.8–4.8			EbpA (1), EbpC (2), horse1 (7), horse2 (1), Peru6 (4), BJH-1 (1), HBH-1 (1), SXH-1 (1)
Stallion	9/186 (4.8)	1.7–8.0	1.407	>0.05	horse1 (2), horse2 (2), CAF1 (1), Peru6 (1), TypeIV (1), HBH-1 (1), BJH-1 (1)
Mare	19/350 (5.4)	3.0–7.8			horse1 (12), EbpA (1), EbpC (2), Peru6 (3), SXH-1 (1)
Castrated horse	2/85 (2.4)	0–5.6			horse1 (2)

**FIGURE 2:**
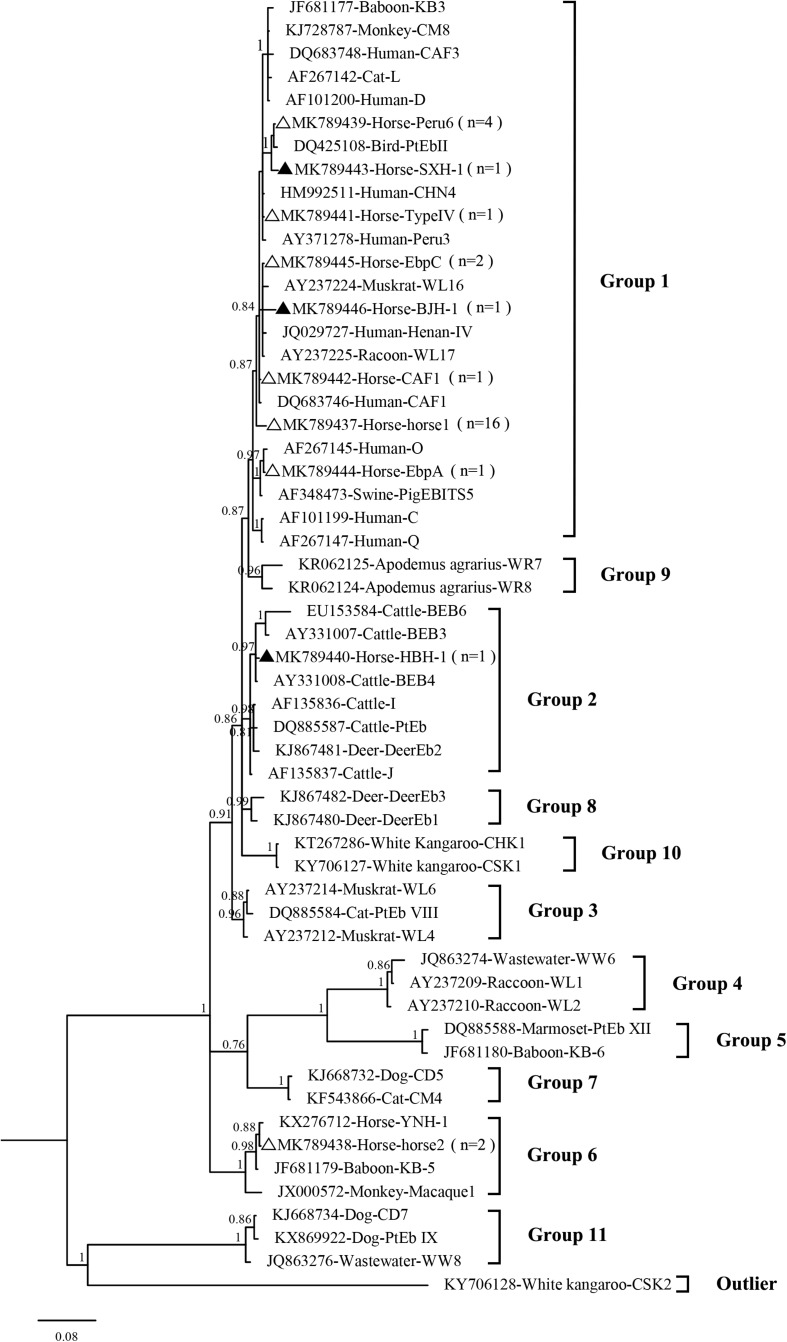
Phylogenetic tree based on Bayesian analysis of the ITS sequences. Statistically significant posterior probabilities are indicated on the branches. The specimen names include the GenBank accession numbers followed by the host and genotype designation. The *E. bieneusi* genotype CSK2 (KY706128) from the white kangaroo was used as the outlier. Known and new genotypes are indicated by hollow and filled triangles, respectively.

In the present study, 10 *E. bieneusi* genotypes were identified in racehorses, including seven known genotypes (EbpC, EbpA, Peru6, horse1, horse2, CAF1, and TypeIV) and three novel genotypes (HBH-1, SXH-1, and BJH-1). The three novel genotypes, HBH-1, SXH-1, and BJH-1, were closely related to genotypes BEB4, Peru6, and Henan-I, with one, two, and four single nucleotide polymorphisms, respectively. Genotype HBH-1 had one nucleotide substitution (T→C) relative to genotype BEB4. Genotype SXH-1 had two nucleotide substitutions (G→A and C→T) relative to genotype Peru6. Genotype BJH-1 had four nucleotide substitutions (G→A, G→A, T→C, and T→C) relative to genotype Henan-I. Among the ten genotypes, horse1 (53.3%, 16/30) dominated, followed by Peru6 (13.3%, 4/30), horse2 (6.7%, 2/30), EbpC (6.7%, 2/30), and each of the remaining six genotypes (3.3%, 1/30). Our phylogenetic analysis revealed that the EbpC, EbpA, TypeIV, Peru6, CAF1, horse1, SXH-1, and BJH-1 genotypes clustered into Group 1, HBH-1 into Group 2, and horse2 into Group 6 (**Figure 2**). Among these genotypes, EbpA, EbpC, Peru6, and TypeIV have been identified in a wide range of hosts, including humans, non-human primates, pets, domestic animals and wild animals (Santín and Fayer, 2011; Wang et al., 2018). Genotype horse1 has been found in horses and non-human primates, genotype horse2 has been found in horses, black bears and squirrels, and genotype CAF1 has been found in horses, pigs, goats, deer and humans (Breton et al., 2007; Jeong et al., 2007; Wagnerová et al., 2012; Deng et al., 2016a, 2017; Qi et al., 2016; Shi et al., 2016; Zhong et al., 2017). Genotypes EbpC and Type IV have been detected in humans in several regions of China (Karim et al., 2014; Yang et al., 2014; Liu et al., 2017; Wang et al., 2017). Future studies should evaluate the molecular epidemiology of *E. bieneusi* genotypes in horses and other animals to better elucidate its transmission dynamics.

Possible genotypic differences in *E. bieneusi* dominance in horses in different geographic areas should also be determined. Dominant genotypes were observed, such as horse1 in Colombia, genotype D in the Czechia, horse2 in Sichuan and Yunnan, China, and the dominant genotypes EbpA and EbpC in Xinjiang, China (Santín et al., 2010; Wagnerová et al., 2012; Laatamna et al., 2015; Deng et al., 2016b; Qi et al., 2016; Tables 2, 3). Therefore, *E. bieneusi* infections in horses likely differ regionally.

## Conclusion

Our results revealed a relatively low occurrence of *E. bieneusi* in racehorses. Ten *E. bieneusi* genotypes were identified, with horse1 being predominant. The observations of five genotypes (EbpC, EbpA, Peru6, TypeIV, and CAF1) in humans as well as three novel genotypes (BGH-1 and SXH-1 in Group 1 and HBH-1 in Group 2) suggest the possibility that racehorses may transmit *E. bieneusi* to humans.

## Data Availability

The datasets generated for this study can be found in the GenBank under the accession numbers MK789437–MK789446.

## Ethics Statement

The Ethics Review Committee of Henan Agricultural University reviewed and approved this research under the approval number IRC-HENAU-20160225. The equestrian club owners granted permission for specimen collection. Animals were handled in accordance with the Animal Ethics Procedures and Guidelines of the People’s Republic of China.

## Author Contributions

MQ and LZ designed the study. AZ, ZW, YP, and YnZ collected and analyzed the specimens. DL and YxZ analyzed the data. AZ, MQ, and LZ wrote the manuscript. All authors read and approved the final manuscript.

## Conflict of Interest Statement

YP was employed by the company Equivets, China. The remaining authors declare that the research was conducted in the absence of any commercial or financial relationships that could be construed as a potential conflict of interest.

## Footnotes

1 https://www.niaid.nih.gov/research/emerging-infectious-diseases-pathogens

2 http://technelysium.com.au

3 https://www.ncbi.nlm.nih.gov/

4 http://clustal.org/

5 https://www.ncbi.nlm.nih.gov/genbank/

6 http://mrbayes.sourceforge.net/

7 http://www.softpedia.com/get/Science-CAD/Modeltest.shtml

8 https://www.softpedia.com/get/Science-CAD/FigTree-AR.shtml
